# The Influence of Structural Constraints and Configurations on Corrosion-Induced Cracking in Reinforced Concrete Based on the Phase-Field Method

**DOI:** 10.3390/ma18174199

**Published:** 2025-09-07

**Authors:** Pengfei Zhang, Lingye Leng, Wenqiang Xu, Sheng Qiang, Hui Wang, Ziang Zhao

**Affiliations:** 1 Department of Architectural Engineering, Jiangxi Water Resources Institute, Nanchang 330013, China; zhangpengfei812@126.com; 2 College of Urban Construction, Jiangxi University of Technology, Nanchang 330022, China; 3 College of Water Conservancy and Hydropower, Hohai University, Nanjing 210098, China; sqiang209@163.com; 4 Henan River Diversion Project Co., Ltd., Zhengzhou 450000, China; wanghui20082010@126.com (H.W.); ziang8156@126.com (Z.Z.)

**Keywords:** phase-field method, reinforced concrete, corrosion-induced cracking, constraint conditions, structural configuration, crack propagation, concrete cover, durability assessment

## Abstract

Corrosion-induced cracking of reinforced-concrete (RC) covers is well known, yet key knowledge gaps persist. Most studies isolate uniform corrosion or a single non-uniform corrosion pattern and ignore the effects of boundary restraint and structural configurations, leading to inaccurate predictions of cracking thresholds and crack propagation patterns. This study systematically investigates the influence mechanisms of constraint conditions and structural configurations on corrosion-induced cracking behavior using the phase-field model. The results indicate that the non-uniformity of steel corrosion is a critical factor governing cover cracking. As the corrosion non-uniformity coefficient increases, the critical corrosion level exhibits a monotonic decreasing trend—from 0.95% to 0.15% under strong constraints and from 0.52% to 0.15% under weak constraints. Concurrently, the crack morphology evolves from a single radial crack to a wedge-shaped crack oriented toward the peak corrosion side. The influence of constraint conditions is dualistic, while strong constraints enhance the failure threshold, their mitigating effect diminishes markedly under highly non-uniform corrosion. The critical corrosion threshold for eccentrically arranged corner reinforcement is significantly lower than that for centrally arranged reinforcement; the corrosion angle only induces slight crack deflection and minor threshold fluctuations; and the curved top section, due to its weaker equivalent constraint, exhibits inferior crack resistance compared to the linear top section. Three-dimensional analysis reveals a pronounced longitudinal discreteness effect, which not only substantially elevates the critical corrosion threshold but also leads to diverse spatial failure modes. This work links rust-expansion eigen-displacement to crack propagation within a unified phase-field framework, providing materials-level criteria for evaluating corrosion tolerance and guiding the design of cover materials and reinforcement layouts to enhance durability.

## 1. Introduction

Reinforced-concrete (RC) structures are widely used in infrastructure such as bridges, buildings, municipal works, and harbor engineering, with their long-term performance directly impacting societal operational safety and sustainable economic development. In service environments, chloride ion ingress and carbonation can lead to steel depassivation and subsequent corrosion. The volumetric expansion of corrosion products (approximately 2–6 times that of the original steel) induces circumferential tensile stresses in the concrete cover, triggering cracking. Once cracks penetrate the surface, harmful agents accelerate ingress, forming a “crack–corrosion” positive feedback loop that rapidly degrades durability [1,2]. Compared to idealized uniform corrosion, the more prevalent unilateral or localized non-uniform corrosion in engineering practice can induce early cracking at lower mass loss and alter crack morphology and topology [3,4,5]. Therefore, accurately determining the corrosion-induced cracking threshold and modeling crack evolution are crucial for service-life assessment and structural detailing optimization.

Current research approaches primarily include experimental observation, analytical modeling, and numerical simulation. Laboratory-accelerated corrosion tests and field monitoring can reveal typical crack patterns—from radial penetration to wedge-shaped spalling—providing relatively realistic durability predictions [6]. However, environmental corrosion tests are time-consuming, requiring extended periods to yield results, and are costly. Additionally, uncertainties in environmental conditions and specimen placement may introduce variability in test outcomes. Analytical and empirical models, often based on assumptions such as thick-walled cylinders and elastic fracture mechanics, can approximate critical corrosion levels under specific conditions. However, their applicability to the entire crack initiation–propagation process and complex boundary or non-uniform corrosion effects remains limited [7]. Numerical simulations, with their high efficiency and controllability, have emerged as an effective tool for predicting structural corrosion progression and residual service life [8,9].

Recent works further demonstrate that durability is strongly conditioned by structural type and detailing, which regulate transport paths, restraint, and stress redistribution during corrosion. State-of-the-art reviews underline standardized crack-control approaches under imposed strains and the debated role of crack width during propagation [10,11,12]. Member geometry, reinforcement layout (bar diameter/spacing, multi-directional bars, transverse reinforcement), and cover systems shift crack initiation thresholds and crack morphology: multi-directional reinforcement can reorient cracks and even trigger cracking along non-corroding bars; transverse reinforcement delays rust-expansion cracking—especially for corner bars at small spacing—while cover thickness/quality influences both initiation and surface crack widths [13,14,15]. In beams, explicit 3D modeling of stirrups under non-uniform attack shows earlier serviceability limit states and more severe cracking than under uniform corrosion, with confinement decaying away from stirrup locations [16]. At the interface scale, non-destructive monitoring reveals circumferential non-uniform strains and pit distributions that align with crack initiation and growth [17,18].

Service conditions further modulate damage evolution. Impressed-current acceleration can underestimate cracking relative to natural wetting–drying regimes, and sustained load reshapes crack patterns while accelerating crack-width growth at serviceability [19,20]. From a performance standpoint, maximum surface crack width correlates more strongly with residual bond capacity than nominal corrosion loss, particularly under non-uniform corrosion [21]. Corrosion-induced cover spalling initiates from inclined cracks rooted at corroded bars and evolves with corrosion loss, with implications for shear behavior in beams; integrated simulations of chloride-induced corrosion corroborate these trends at the structural scale [22,23].

However, crack propagation is a typical discontinuous problem. While traditional cohesive zone and fracture models can partially describe cracking characteristics, they often rely on predefined crack paths or weakened zones. When dealing with concrete cover damage caused by steel corrosion—particularly in scenarios involving three-dimensional or complex crack propagation—these methods lack effective handling capabilities [24,25,26]. In contrast, the phase-field fracture method diffuses sharp cracks, enabling unified characterization of crack initiation, propagation, branching, and merging without explicit path tracking. It also facilitates coupling with multi-physics processes, leading to rapid development in quasi-brittle fracture research in recent years [27,28]. Based on the unified phase-field theory, Wu et al. [29] proposed a unified phase-field model capable of accurately describing crack propagation in quasi-brittle materials like concrete, which has been widely applied. Adaptive meshing and three-dimensional implementations have further improved computational accuracy and efficiency for complex crack problems [30,31]. For concrete softening behavior, the PF_CZM (Phase Field–Cohesive Zone) model more realistically captures crack nucleation and softening stages, having been validated in studies on size effects and concrete fracture simulations [29].

The phase-field method has also been applied to simulate corrosion-induced cracking, demonstrating good agreement with experimental and engineering observations. Francesco Freddi et al. [32] developed a brittle fracture model coupling concrete carbonation with phase-field fracture to depict the depassivation–corrosion process of steel and subsequent cover cracking. Congjie Wei et al. [33] formulated a multi-field (hydro-chemo-mechanical) coupled phase-field framework, clarifying the critical roles of concrete fracture toughness and permeability in corrosion-induced crack evolution. Xurui Fang et al. [34,35] proposed computational frameworks coupling diffusion–corrosion and corrosion–phase-field fracture, successfully reproducing cover damage under non-uniform corrosion and aligning with experimental results. Furthermore, some scholars, after establishing phase-field models for steel corrosion, systematically investigated the influence of parameters such as cover thickness, rebar diameter and spacing, and rebar quantity on corrosion-induced damage responses [36,37,38,39].

However, existing studies predominantly focus on uniform corrosion or a single type of non-uniform corrosion, lacking comprehensive comparisons and analyses of multiple corrosion patterns. Moreover, they often fail to systematically account for the coupling effects between engineering factors—such as external constraints (e.g., simply supported specimens, fixed boundaries, free edges), rebar distribution and eccentricity within cross-sections, and specimen geometry (e.g., surface curvature)—and corrosion morphology, leading to deviations in predicting cracking thresholds and crack patterns. Most numerical studies remain idealized two-dimensional analyses, treating longitudinal corrosion as a uniform field while neglecting three-dimensional non-uniform envelopes and end effects, potentially resulting in overly conservative or unconservative assessments of corrosion-induced cracking [40]. These limitations render the simulated cover damage modes relatively simplistic, inconsistent with the diverse crack topologies and failure modes observed in real-world engineering.

To address these issues, this study uses a PF_CZM framework for corrosion-induced cracking. The rust expansion effect is characterized using equivalent interfacial normal displacement, while a power-law function describes circumferential non-uniform corrosion morphology. The influences of external constraints, rebar position, corrosion angle, top-surface geometry, and three-dimensional longitudinal non-uniform corrosion on cover cracking thresholds and crack patterns are investigated. The paper is organized as follows: Section 2 introduces the phase-field model and numerical implementation, corrosion morphology and parameter calibration, unified evaluation metrics, and the numerical test matrix; Section 3 presents simulation results, quantifying the effects of individual and combined factors on critical corrosion levels and crack topology; Section 4 discusses key mechanisms, evaluates the relative significance of each factor, and identifies study limitations and future research directions; Section 5 summarizes the main conclusions.

## 2. Methodology

### 2.1. Phase-Field Method

#### 2.1.1. Theoretical Framework

The phase-field method (PFM) for fracture modeling has evolved into a well-established theoretical framework. Building upon the unified phase-field theory by Wu Jianying et al. [41,42] and prior studies [31,43], a phase-field model for simulating steel corrosion-induced damage was developed in this work.

Drawing from image segmentation theory [44], the crack region can be represented as a volume fraction [45]:

(1) $$\underset{\mathrm{Sharp}\;\mathrm{crack}}{\underset{︸}{A_{s}=\int_{S}dA}}=\underset{\mathrm{Regularized}\;\mathrm{crack}}{\underset{︸}{\int_{B}\gamma (d;\nabla d)=A_{d}(d)}}$$

Here, S denotes the sharp crack surface, while B represents the smeared crack zone, where the sharp crack S is regularized into a finite-width region B. The crack phase field d characterizes the damage state, with d=0 indicating an intact material and d=1 a fully broken state. The crack density functional ∇d, as formulated by Ambrosio et al. [46], is given by

(2) $$\left\{\begin{array}{l} \gamma (d;\nabla d)=\frac{1}{c_{a}}\left[\frac{1}{l}\alpha (d)+l{\left|\nabla d\right|}^{2}\right]\\ c_{a}=4\int_{0}^{1}\sqrt{\alpha (\beta )}d\beta \end{array}\right.$$

The crack geometric function α(d), a monotonically increasing function of d with α in [0,1], is typically defined as

(3) $$\alpha (d)=\xi d+(1-\xi )d^{2}$$

where ξ is an adjustable parameter [0,2].

Enforcing the laws of thermodynamics and crack irreversibility, the phase-field evolution in the domain follows [42]:

(4) $$\left\{\begin{array}{l} \nabla \cdot q+Q\;(\varepsilon ,d)\leq 0,\;\mathrm{in}\;Β\\ q\cdot n_{B}\geq 0,\;\mathrm{on}\;Β \end{array}\right.$$

Here, q and Q (ε,d) represent the phase-field flux and source term, respectively, and $n_{B}$ is the crack surface normal. These terms are expressed as

(5) $$\left\{\begin{array}{l} q:=\frac{2l}{c_{a}}G_{f}\nabla d\\ Q\;(\varepsilon ,d)=-\omega^{\prime }(d)\bar{Y}-\frac{1}{c_{a}l}G_{f}\alpha^{\prime }(d) \end{array}\right.$$

where $G_{f}$ is the material fracture energy, ε is the strain tensor, $\omega^{\prime }(d)$ the derivative of the energy degradation function, and $\bar{Y}$ the effective energy release rate. The degradation function ω(d) and $\bar{Y}$ are defined by

(6) $$\left\{\begin{array}{l} \bar{Y}:=\frac{\partial \psi }{\partial \omega }\\ \omega (d)=\frac{{\left(1-d\right)}^{r}}{{\left(1-d\right)}^{r}+a_{1}d\cdot (1+a_{2}\cdot d+a_{3}\cdot d^{2})} \end{array}\right.$$

with ψ denoting the strain energy density and r, $a_{1}$, $a_{2}$, and $a_{3}$ being experimentally determined coefficients.

Concurrently, the displacement field (u) satisfies the equilibrium equation:

(7) $$\left\{\begin{array}{l} \nabla \cdot \sigma +b=0,\;\mathrm{in}\;\Omega \\ \sigma \cdot n=t,\;\mathrm{on}\;\partial \Omega \end{array}\right.$$

where σ is the stress tensor, b the body force, and t the boundary traction.

#### 2.1.2. Numerical Implementation

The governing equations Equations (4) and (7) are transformed into weak forms:

(8) $$\left\{\begin{array}{l} \int_{\Omega }b\cdot \delta udV+\int_{\partial \Omega }t\cdot \delta udA=\int_{\Omega }\sigma :\nabla \delta udV\\ \int_{B}Q\delta ddV-\int_{B}q\cdot \nabla \delta dV\leq 0 \end{array}\right.$$

Using finite element discretization, the displacement field, phase field, and their gradients are approximated as

(9) $$\begin{array}{l} u(x)=Na,\;\varepsilon (x)=Ba\\ d(x)=\bar{N}\bar{a},\;\nabla d=\bar{B}\bar{a} \end{array}$$

Here, a and $\bar{a}$ are nodal degrees of freedom for the displacement and phase field, N and $\bar{N}$ are shape functions, and B and $\bar{B}$ are gradient matrices (see [47] for details).

The system is solved incrementally, dividing the load/time step [0,T] into M sub-steps, like m=0,1,…,M−1. For each sub-step $\Delta t:=t_{m+1}-t_{m}$, state variables are updated iteratively from known values at $t_{m}$.

To enforce crack irreversibility, the historical field variable H replaces $\bar{Y}$:

(10) $$H=\max_{m\in [0,T]}(\bar{Y_{0}},{\bar{Y}}_{m+1})\to Q(\varepsilon ,d)=-\omega^{\prime }(d)H-\frac{G_{f}}{c_{a}l}\alpha^{\prime }(d)$$

where Y0¯=f2t2E0 is the energy release threshold for crack initiation.

This transforms the phase-field inequality into

(11) $$\left\{\begin{array}{l} r=f^{ext}-\int_{\Omega }B^{T}\sigma dV=0\\ \bar{r}=\int_{B}{\bar{N}}^{T}QdV-\int_{B}{\bar{B}}^{T}qdV=0 \end{array}\right.$$

The staggered iterative scheme [48,49] proceeds as follows for each load increment:

Displacement Update: Fix $\bar{a}={\bar{a}}^{\left(k-1\right)}$ and solve for $a^{k}$ and $\sigma^{k}$:

(12) $$\left\{\begin{array}{l} \underset{︸}{K_{uu}\delta a^{k}=\int_{\Omega }B^{T}\omega (\bar{a})DBdV\delta a^{k}}=\underset{︸}{f^{ext}-\int_{\Omega }B^{T}\bar{\sigma }dV=r}\\ \bar{\sigma }=\omega (\bar{a})\cdot DBa^{k-1}\\ a^{k}=a^{k-1}+\delta a^{k}\\ \sigma_{k}=DBa^{k} \end{array}\right.$$

Phase-Field Update: Fix $a=a^{k}$ and solve for ${\bar{a}}^{k}$:

(13) $$\left\{\begin{array}{l} \underset{︸}{K_{dd}\delta {\bar{a}}^{k}=\int_{B}\left[{\bar{N}}^{T}\left(-\frac{\partial Q}{\partial d}\right)\bar{N}+\frac{2l}{c_{a}}G_{f}{\bar{B}}^{T}\bar{B}\right]dV\delta {\bar{a}}^{k}}=\underset{︸}{\int_{B}{\bar{N}}^{T}QdV-\int_{B}{\bar{B}}^{T}qdV=\bar{r}}\\ Q(\sigma_{k},{\bar{a}}^{\left(k-1\right)})=-\omega^{\prime }({\bar{a}}^{\left(k-1\right)})H^{k}-\frac{G_{f}}{c_{a}l}\alpha^{\prime }({\bar{a}}^{\left(k-1\right)})\\ H^{k}=\max\left(\frac{\max(f_{t},{\left[\sigma_{k}\right]}^{1})}{2E_{0}},H^{\left(k-1\right)}\right)\\ {\bar{a}}^{k}={\bar{a}}^{\left(k-1\right)}+\delta {\bar{a}}^{k} \end{array}\right.$$

Convergence Check: Terminate if ${\left\|\delta \bar{a^{k}}\right\|}_{2}<1\times 10^{-5}$; otherwise, repeat.

The MATLAB(2023a)-based phase-field package (PFM) [31,43,50] has been extended to include corrosion-induced damage calculations and is available upon request.

### 2.2. Numerical Experiments

#### 2.2.1. Computational Model and Parameters

PF_CZM was employed to simulate the fracture behavior of concrete induced by steel corrosion. Compared to conventional AT1 and AT2 models, this model more accurately characterizes crack nucleation in concrete and the strain-softening behavior post-initiation, with predicted crack propagation paths and corrosion-induced displacement fields showing good agreement with experimental results. The softening coefficient adopted the recommended value from Wu Jianying et al. [51], and specific material parameters are listed in Table 1. It should be noted that parameters such as $c_{a}$, $a_{1}$, $a_{2}$, $a_{3}$, m, and ξ in the table are fitting parameters for the concrete constitutive model. The mechanical properties of concrete, referenced from engineering measurements and the literature [29], are provided in Table 2.

Studies indicate that steel corrosion morphology significantly influences concrete cover cracking [52]. In engineering practice, steel corrosion primarily manifests in three typical modes: (1) Uniform corrosion: Induced by concrete carbonation, leading to uniform corrosion across the entire cross-section [52]. (2) Non-uniform corrosion: Caused by chloride ion ingress, resulting in localized corrosion on one side of the reinforcement [4,5]. (3) Pitting corrosion: Localized corrosion pits formed at vulnerable regions of the steel surface due to aggressive ion penetration [53].

To quantitatively characterize these corrosion patterns, a mathematical model based on a power-law function was developed to accurately describe the gradual transition of the corrosion front. Let $d_{\theta }$ denote the corrosion loss at different cross-sections of the rebar, with $d_{c}$ representing the maximum loss. In polar coordinates, the corrosion profile on the right side of the rebar can be expressed as

(14) $$d_{\theta }={\left(b_{2}\cdot (\theta +a_{2})\right)}^{c_{2}}\cdot d_{c}$$

The left-side profile is obtained via mirror symmetry. Based on experimental data from Jang et al. [54], the parameter α (corrosion coefficient) for different corrosion modes falls within the following ranges:

(15) $$\left\{\begin{array}{lllll} \alpha =1 & a_{2}=1 & b_{2}=1 & c_{2}=0 & \theta \in \left[-\pi ,\pi \right]\\ \alpha =2 & a_{2}=\frac{\pi }{2} & b_{2}=\frac{1}{\pi } & c_{2}=1.5 & \theta \in \left[-\frac{\pi }{2},\frac{\pi }{2}\right]\\ \alpha =4 & a_{2}=0 & b_{2}=\frac{2}{\pi } & c_{2}=1.3 & \theta \in \left[0,\frac{\pi }{2}\right]\\ \alpha =8 & a_{2}=-\frac{\pi }{4} & b_{2}=\frac{4}{\pi } & c_{2}=1.8 & \theta \in \left[\frac{\pi }{4},\frac{\pi }{2}\right] \end{array}\right.$$

Considering the expansion effect of corrosion products, with a volume increase approximately 2–6 times the initial volume [55], an intermediate value of 3 times the expansion ratio is adopted in the calculations for comparable analysis under a unified expansion assumption, i.e., the maximum corrosion-induced expansion displacement. To evaluate sensitivity, a parameter sweep of the expansion coefficient (k) is conducted without altering other parameters. The results indicate that the critical corrosion level decreases approximately proportionally with increasing expansion ratio. For instance, under the representative condition of α = 1, the critical values for k = 3 and k = 6 are approximately 0.52% and 0.26%, respectively, while the crack initiation location and dominant path remain consistent. Therefore, the comparative conclusions drawn in this study remain robust across different expansion coefficients.

The corresponding radial displacement field $d_{a}$ is given by

(16) $$d_{a}={\left(b_{2}\cdot (\theta +a_{2})\right)}^{c_{2}}\cdot d_{\max}={\left(b_{2}\cdot (\theta +a_{2})\right)}^{c_{2}}\cdot \left(3d_{c}\right)$$

**Figure 1:**
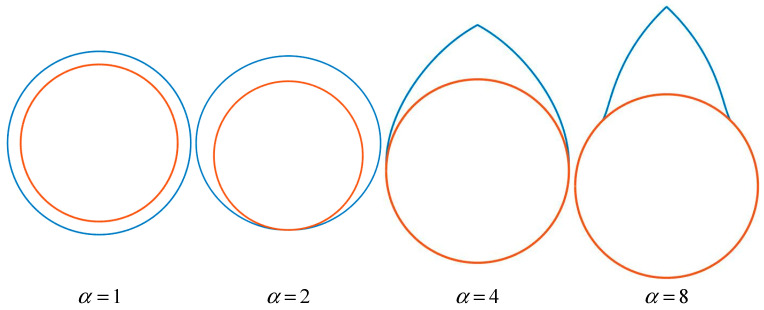
Different types of non-uniform corrosion morphology (Red is the initial steel surface; Bule is the corrosion products).

Figure 1 visually compares the initial (red) and corroded (blue) profiles, illustrating the morphological evolution under a varying corrosion coefficient α. Here, α=1 corresponds to uniform corrosion, while increasing α enhances non-uniformity.

#### 2.2.2. Constraint Conditions

To investigate the influence of external constraints on corrosion-induced cracking, a plane-strain computational domain of 150 mm × 150 mm was adopted, containing an initial reinforcement interface with radius r = 9 mm and a concrete cover thickness of 20 mm. The rust expansion effect was simulated by applying an angle-dependent equivalent normal displacement field at the initial interface $\Gamma_{i}$, without explicitly modeling the steel reinforcement and corrosion products. The displacement field was determined according to different corrosion conditions of the reinforcement using Equation (16). Only normal displacement was constrained at the interface, while tangential movement remained unconstrained.

**Figure 2:**
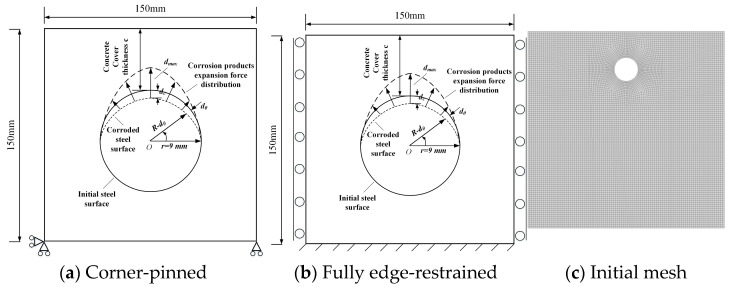
Different constraint types and initial mesh (The arrow indicates the corrosion depth of the steel bar, the triangle and circle together are the hinge support constraints, and the dotted line shows the surface of the corroded steel bar).

To ensure the accuracy of the computational results, the initial mesh element size (h) is set to approximately 1.5 mm, as shown in Figure 2c. An adaptive mesh refinement technique is employed, whereby the element size (h) within the fracture process zone is adaptively refined to 0.5 mm. The phase-field regularization width l is set to 4 mm, resulting in a ratio of h/l = 1/8. For the adopted PF_CZM model, studies by Wu Jianying et al. [51] have demonstrated that numerical results are virtually unaffected by the mesh element size provided the condition h/l ≤ 1/5 is satisfied. The element size used in this study meets the recommended requirement of h/l ≤ 1/5, thereby fulfilling the criteria for numerical stability. Subsequent calculations employ a similar mesh element size.

Two boundary conditions were applied to the external boundary ∂Ω: (a) Simply supported corner constraints: Natural free boundaries were employed, with minimal displacement constraints applied at two corner points to eliminate rigid-body translation and rotation, as shown in Figure 2a; (b) Peripherally fixed constraints: Normal displacement constraints were imposed on the left and right boundaries, while the bottom boundary was fully fixed, as illustrated in Figure 2b. For both cases, displacement-controlled quasi-static incremental loading was implemented. The displacement $d_{c}$ was monotonically increased from 0 until instability occurred, with the displacement and phase fields updated alternately through iterative calculations. Crack irreversibility was ensured by the historical field variable. All material and numerical parameters remained identical except for the boundary constraints. The corrosion coefficient α was varied from 1 to 8 to analyze the effect of constraint intensity on concrete cover damage under different corrosion coefficients.

Experimental and modeling studies indicate that strong external restraint increases the corrosion demand at crack initiation and promotes multi-cracking or spalling, whereas free boundaries tend to trigger earlier cracking with predominantly single-crack patterns [12,56]. Accordingly, we consider two representative boundary scenarios—corner-pinned (quasi-free) and fully edge-restrained (strong restraint)—to span the practical restraint spectrum and numerically examine how restraint intensity influences the crack initiation threshold and crack morphology. In practice, free top edges of exterior wall slabs and regions around slab openings are closer to quasi-free boundaries, while beam–slab–wall junctions and beam–shear–wall connections are closer to strongly restrained conditions.

#### 2.2.3. Reinforcement Distribution Position

**Figure 3:**
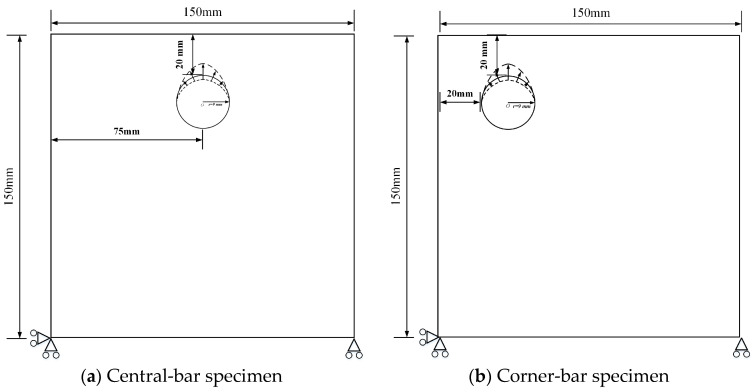
Different reinforcement distribution positions (The arrow indicates the corrosion depth of the steel bar, the triangle and circle together are the hinge support constraints, and the dotted line shows the surface of the corroded steel bar).

To evaluate the modulating effect of reinforcement spatial distribution in the concrete cover on corrosion-induced cracking, an eccentric single-rebar configuration (Figure 3b) was established under simply supported constraints (Figure 3a). In the eccentric single-rebar case, the same 150 mm × 150 mm plane-strain computational domain and circular initial interface with radius r = 9 mm were maintained. The rebar center was offset leftward relative to the plate center, resulting in a left-side cover thickness of 20 mm while keeping all other parameters and corrosion displacement field definitions unchanged. The corrosion coefficient α was varied from 1 to 8 to analyze the influence of rebar eccentricity on cover damage under different corrosion coefficients.

In engineering practice, eccentric reinforcement layouts are common due to reduced cover near the slab, wall edges, balcony cantilevers, or bar misplacement along beam sides. Empirical evidence shows that a thinner cover and proximity to corners significantly lower the cracking threshold of reinforced concrete and alter crack trajectories [57]. Therefore, we compare centered and eccentric bar configurations to numerically assess how reinforcement position modulates the crack initiation threshold and crack morphology.

#### 2.2.4. Corrosion Angle of Reinforcement

**Figure 4:**
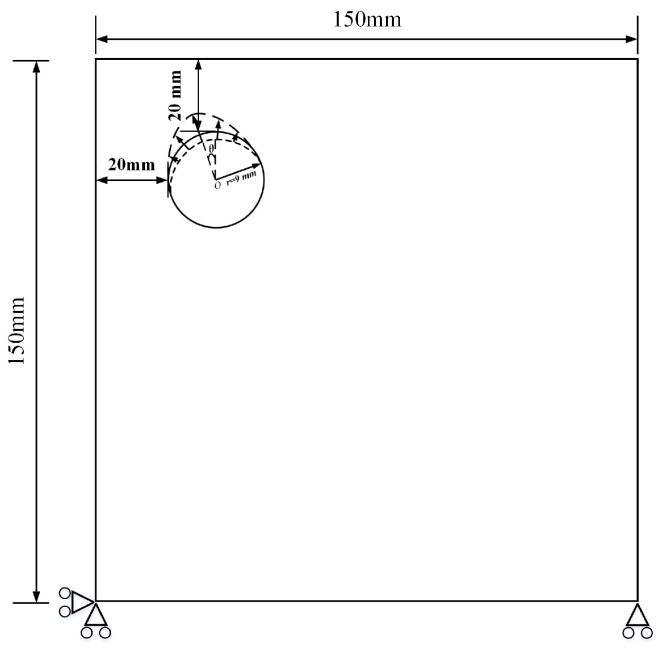
Different corrosion angles (The arrow indicates the corrosion depth of the steel bar, the triangle and circle together are the hinge support constraints, and the dotted line shows the surface of the corroded steel bar.).

To investigate the effect of corrosion front orientation along the circumferential direction on cover cracking, the phase angle of the interfacial corrosion displacement field in polar coordinates was modified while maintaining the simply supported corner constraints and eccentric rebar configuration (Figure 4). The computational domain and material parameters remained consistent with previous settings.

In the numerical implementation of rotated corrosion angles, the reference non-uniform corrosion displacement distribution $d_{a}(\theta )$ was rotated by φ degrees along the circumference, expressed as $d_{a}(\theta )=d_{a}(\theta -\varphi )$. Here, φ denotes the polar angle from the node to the rebar center, with the positive y-axis as 0° and counterclockwise as positive. This approach only alters the angle of the corrosion pattern without changing its magnitude or non-uniformity degree.

The computational cases considered φ ∈ {0°, 5°, 10°, 20°} and non-uniformity coefficients α ∈ {2, 4, 8}. Note that when α = 1, the distribution becomes constant and rotation has no effect; hence, this case was excluded. Other meshing and convergence parameters matched the previous settings to analyze how rebar corrosion angles affect cover damage under different corrosion coefficients.

In field conditions, prevailing sea-spray exposure, wind-driven rain, or flow-induced wetting can produce directional bias in chlorides and moisture, leading to a circumferential “phase shift” of corrosion around the bar. Studies report directionally biased non-uniform pitting or semi-ring corrosion along the bar circumference, with the phase location affecting crack initiation sites and surface crack orientation [58,59]. Hence, while keeping corrosion amplitude and non-uniformity unchanged, we rotate only the circumferential phase of the corrosion distribution to isolate the effect of directional bias on initiation and crack morphology.

#### 2.2.5. Structural Configurations

**Figure 5:**
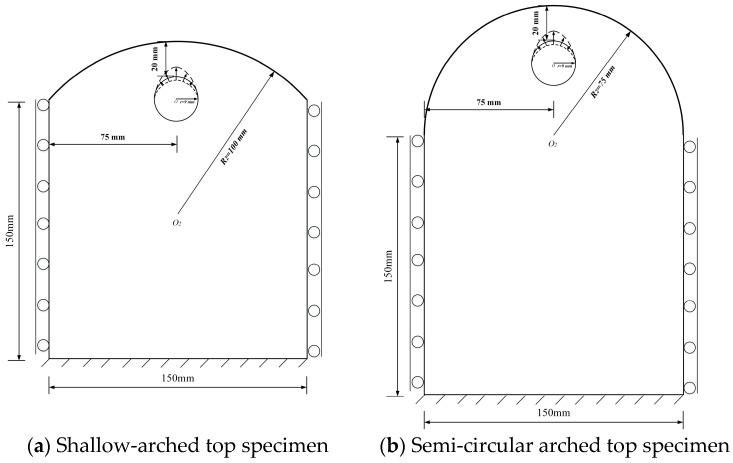
Different structural configurations (The arrow indicates the corrosion depth of the steel bar, the triangle and circle together are the hinge support constraints, and the dotted line shows the surface of the corroded steel bar).

To evaluate the influence of top-surface geometry on corrosion-induced cracking, only the geometric configuration of the specimen’s top surface was modified while maintaining identical material parameters and other settings as described previously. The computational domain featured a horizontal span of 150 mm with straight left, right, and bottom boundaries. Three top surface configurations were implemented: (1) flat top (baseline), (2) shallow arched top (Figure 5a radius = 100 mm), (3) semi-circular arched top (Figure 5b, radius = 75 mm).

A single circular reinforcement interface with radius r = 9 mm was horizontally positioned at mid-span. To ensure comparability across different structural configurations, the minimum concrete cover thickness between the reinforcement and top surface was consistently maintained at 20 mm at mid-span. Only normal equivalent displacement was applied at the interface, while tangential movement remained unconstrained. The external boundaries employed fully fixed constraints.

The corrosion loading protocol followed the same displacement-controlled approach as in previous sections. To highlight the structural configuration effects, simulations were conducted for the three geometries under corrosion coefficients α ∈ {1, 2, 4, 8}. The corrosion angle was fixed at φ = 0° to specifically analyze the influence of structural configuration on concrete cover damage under varying corrosion coefficients.

Curved roof slabs, transverse camber on bridge decks, soil-covered top slabs, and cornice curvatures are commonplace and align with the geometries investigated here. Prior studies show that structural curvature modifies near-surface stress fields and boundary compliance, which can influence corrosion-driven crack thresholds and crack paths [60]. Therefore, with a constant minimum cover, we vary only the top-surface geometry (while fixing corrosion phase and external restraints) to examine how curved boundaries modulate crack morphology.

#### 2.2.6. Three-Dimensional Corrosion Morphology

To investigate the effect of non-uniform corrosion along the longitudinal direction of reinforcement on concrete cover damage, a 3D model measuring 300 mm (length) × 150 mm (width) × 150 mm (height) was established to better simulate real-world conditions. The coordinate system was defined with x (width), y (height), and z (length) directions. A single reinforcing bar with radius r = 9 mm extended continuously along the z-direction, maintaining a constant 20 mm concrete cover thickness.

Boundary conditions applied strong constraints. Bottom surface (y = 0): Fully constrained (u_x_ = u_y_ = u_z_ = 0). Side surfaces (x = 0 and x = 150 mm): Constrained in x and z directions (u_x_ = u_z_ = 0), free in y direction. All other surfaces (top surface y = 150 mm and end surfaces z = 0 and z = 300 mm): Unconstrained. Material properties, numerical implementation, and convergence criteria remained consistent with previous sections.

It should be noted that the fully fixed bottom boundary in this study is considered as an approximation of service conditions with massive support and sufficient anchorage. The displacement constraints in the x and z directions on the side surfaces are employed to equivalently represent the lateral stiffness provided by adjacent segments of wide-span structural members. This configuration corresponds to a representative region extracted from large-volume components (such as bridge decks or hydraulic pier structures), where lateral displacements are effectively restrained by the overall structural stiffness during service. Consequently, this setup can be regarded as a conservative representation of strong constraint scenarios.

**Figure 6:**
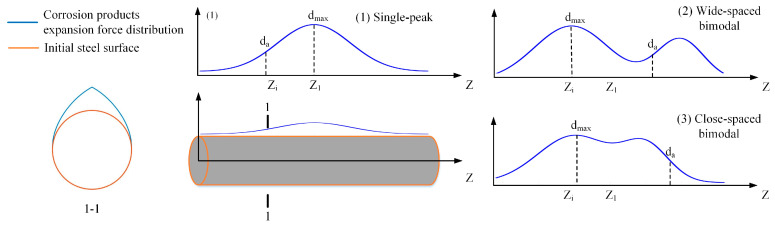
Three-dimensional corrosion morphologies of reinforcement (The arrow indicates the coordinate axis, and the dotted line indicates the expansion depth of corrosion products).

Corrosion was simulated by applying normal equivalent displacement to the initial cylindrical reinforcement surface $\Gamma_{i}$. The circumferential distribution maintained the same non-uniform pattern $d_{a}(\theta )$ with corrosion coefficients α ∈ {1, 2, 4, 8}. Following [53,61], a Gaussian function characterized the longitudinal non-uniformity (Figure 6). The interface normal displacement was expressed as

(17) $$\left\{\begin{array}{l} d_{a}(\theta ,z)={\left(b_{2}\cdot (\theta +a_{2})\right)}^{c_{2}}\cdot \left(3d_{c}\right)\cdot G(z)\\ \Delta L=\sum_{i=1}^{n}\frac{c_{1,i}}{c_{2,i}\sqrt{2\pi }}e^{-{\left(\frac{z-z_{1,i}}{\sqrt{2}c_{2,i}}\right)}^{2}}+c_{3}\;z\in [0,300]\\ G(z)=\frac{\Delta L}{\max(\Delta L)} \end{array}\right.$$

where G(z) ∈ [0,1] represents the longitudinal envelope function, and coefficients $c_{1,i}$, $c_{2,i}$, $z_{1,i}$, and $c_{3}$ describe longitudinal non-uniformity. Three characteristic cases were examined, with corresponding parameters:

(18) $$\left\{\begin{array}{l} \mathrm{Single}\text{-}\mathrm{peak}:\;\\ c_{1,1}=1000\;c_{2,1}=51\;z_{1,1}=150\;c_{3}=1.25\;\\ \mathrm{Wide}\text{-}\mathrm{spaced}\;\mathrm{bimodal}:\;\\ c_{1,1}=1000\;c_{2,1}=51\;z_{1,1}=100\;c_{3}=1.25\\ c_{1,2}=478\;\;\;c_{2,2}=32\;z_{1,2}=250\;\\ \mathrm{Close}\text{-}\mathrm{spaced}\;\mathrm{bimodal}:\;\\ c_{1,1}=1000\;c_{2,1}=51\;z_{1,1}=100\;c_{3}=1.25\;\\ c_{1,2}=478\;\;\;c_{2,2}=32\;z_{1,2}=200 \end{array}\right.$$

The loading and solution procedures matched the 2D cases. Simulations evaluated all three longitudinal corrosion patterns to analyze their 3D effects on concrete cover damage.

In practice, longitudinally localized corrosion frequently occurs in intermittently wetted zones, near drainage outlets, construction joints, and leakage-induced wet areas, consistent with the scenarios modeled here. Evidence shows that corrosion along the bar length exhibits finite, localized extents that can be approximated by single-, broadened-, or double-peaked Gaussian envelopes, which in turn affect crack initiation and propagation patterns [3,53]. Accordingly, we adopt a 3D equivalent displacement field constructed as (circumferentially non-uniform) × (longitudinal Gaussian envelope) and design three representative longitudinal distributions to systematically scan these effects.

### 2.3. Failure Criterion

The characteristic displacement was adopted as the failure criterion for concrete cover damage, defined as the maximum corrosion-induced expansion displacement of the reinforcement when the phase-field value (d) reaches 0.95. This is because, within the phase-field framework, d = 1 indicates complete local fracture. Under the degradation function used in this study, Equation (6), when d = 0.95, the effective stiffness $\omega (d)\cdot E_{0}\approx 6.0\times 10^{-6}\cdot E_{0}$ is reduced to a magnitude of less than 1%, which can be considered as the effective loss of load-bearing capacity, consistent with the operational definition of observable crack formation in engineering practice. This value also falls within the commonly used range of d ∈ [0.90, 0.99] in the phase-field literature. With reference to [40], this paper employs d = 0.95 as the baseline value for the structural failure criterion.

For more intuitive evaluation, a corrosion level parameter (ρ) was introduced [62]:

(19) $$\rho =\frac{M_{loss}}{M_{s}}\times 100\%$$

where $M_{loss}$ represents the mass loss per unit length of reinforcement and $M_{s}$ denotes the original steel mass.

In practical implementation, to unify the phase-field output into “corrosion level”, the characteristic interfacial expansion displacement $d_{\max}$ is extracted at the load step where the phase value d ≥ 0.95 is first satisfied. According to the definition of the non-uniform corrosion model, $d_{\max}$ is substituted into Equation (16) for circumferential integration to obtain the equivalent expansion volume increment. This increment is then divided by the volumetric expansion ratio (k = 3) to determine the metal loss mass $M_{loss}$, based on which the corrosion level ($\rho =M_{loss}/M_{s}$) is calculated. This parameter quantifies the degree of steel corrosion and directly reflects the influence of various factors on the crack resistance of the concrete cover.

### 2.4. Research Program

**Figure 7:**
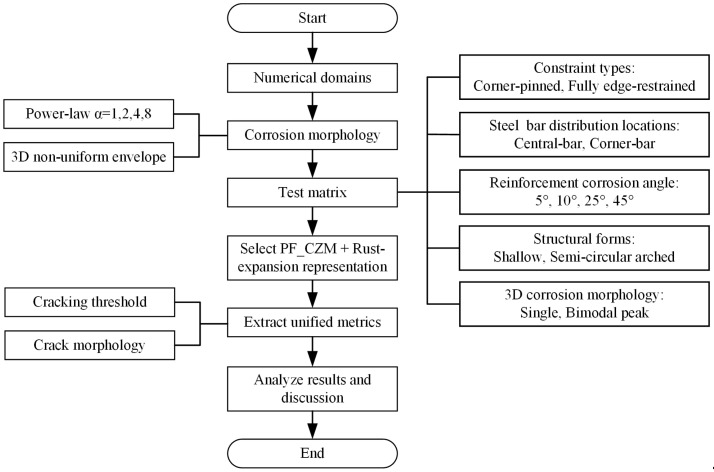
Research program and methodological flowchart.

To make the methodology transparent, we summarize the research program in Figure 7 and follow its top-down pipeline. The flow starts from defining the computational setting, proceeds through corrosion description and the test matrix, and ends with unified metrics and analyses.

We first build 3D numerical domains representing the cover–rebar system used throughout the study. Material parameters and the cohesive fracture response follow the PF_CZM formulation introduced earlier, and the rust expansion is driven by an equivalent interfacial normal displacement.

Corrosion morphology is then prescribed in two dimensions. Circumferential non—uniformity is controlled by a power-law distribution with exponents α ∈ {1, 2, 4, 8}; longitudinal variability adopts a 3D envelope with either a single peak or a bimodal peak to capture local attack and end effects. Parameters are calibrated as described in the corrosion morphology subsection.

Next, the test matrix is assembled by combining the structural and boundary scenarios shown on the right side of Figure 7: constraint types (corner-pinned, fully edge-restrained), bar locations (central-bar, corner-bar), reinforcement corrosion angles (5°, 10°, 25°, 45°), structural forms (shallow, semi-circular arched), and the chosen 3D corrosion morphology (single peak, bimodal peak).

With these inputs fixed, we run simulations using the PF_CZM model with the rust-expansion representation. For each case, we extract unified outputs—such as the cracking threshold and crack morphology—as shown on the left of Figure 7.

Finally, results are synthesized along the central pipeline into the results and discussion stage, where we quantify factor effects and interactions and interpret implications for detailing and service-life assessment. This completes the research program from “Start” to “End” as visualized in Figure 7.

## 3. Results

### 3.1. Non-Uniformity and Constraint Conditions

**Figure 8:**
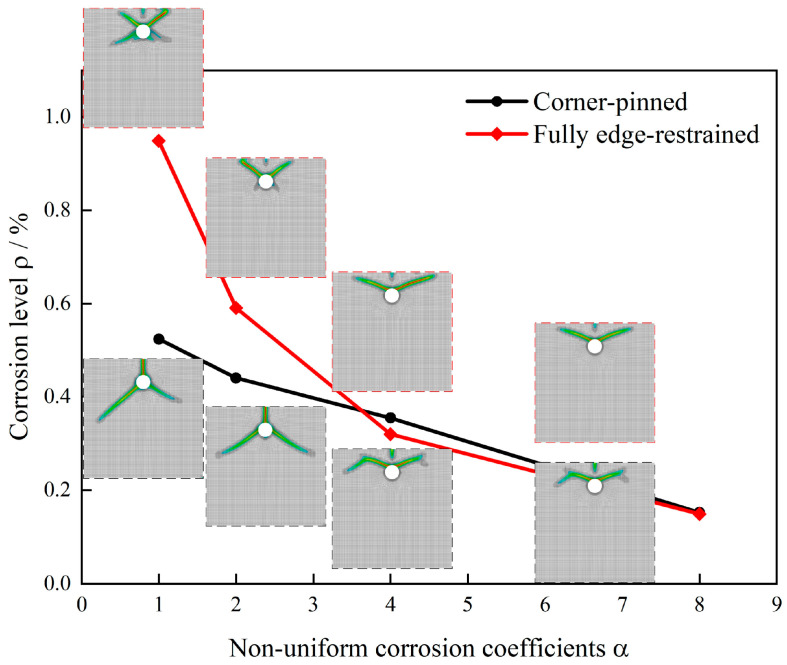
Crack propagation and corrosion levels under different constraint conditions.

Under consistent material and numerical parameters while varying only the external boundary constraints, this study compares crack propagation patterns and corresponding corrosion levels in a concrete cover when the corrosion coefficient α increases from 1 to 8 under two constraint conditions: simply supported corners and fully fixed peripheral boundaries. Figure 8 presents crack evolution contours and ρ−α relationship curves for different corrosion coefficients under both constraint types.

Significant differences in crack propagation morphology emerge due to boundary constraints. For simply supported specimens with greater deformation freedom, non-uniform expansion displacements initially induce principal tensile cracks in the direction of the thinnest cover. At α = 1 (uniform corrosion), cracks propagate symmetrically along the shortest cover path and radially penetrate the surface. As α increases, cracks progressively shift toward the peak corrosion side, ultimately forming wedge-shaped cracks. In contrast, fixed peripheral constraints restrict boundary deformation, causing earlier conversion of interfacial expansion into in-plane tensile stresses and resulting in more complex failure modes. Even under uniform corrosion, wedge-shaped failure occurs; increasing α enlarges the wedge angle and significantly expands the damage zone.

Corrosion level analysis reveals key patterns. Both constraint types exhibit monotonically decreasing critical corrosion levels with an increasing corrosion coefficient, from 0.52% to 0.15% for simply supported constraints and from 0.95% to 0.15% for fixed constraints. This demonstrates that non-uniform corrosion accelerates failure at lower total mass loss by intensifying local expansion gradients and amplifying peak hoop strains. Notably, fixed constraints maintain higher critical corrosion levels than simply supported conditions across most α values, confirming that strong constraints enhance crack resistance. However, the performance gap narrows as α increases, indicating constrained boundaries gradually lose their inhibitory effect under highly non-uniform corrosion.

**Figure 9:**
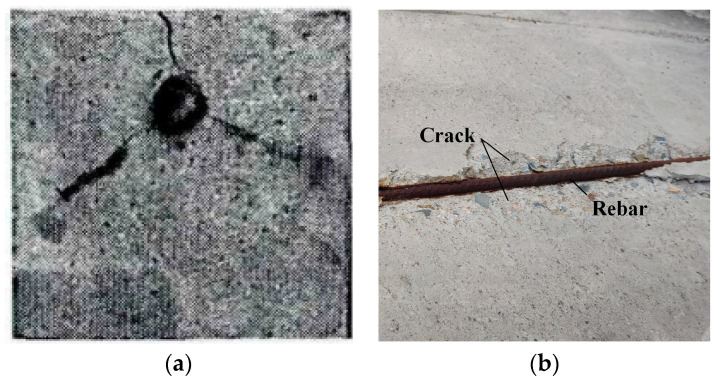
Crack propagation in concrete cover under different constraint conditions. (**a**) Laboratory conditions [63]. (**b**) Engineering conditions [39].

Numerical results align well with empirical observations. Weakly constrained laboratory specimens predominantly develop single radial penetrating cracks (Figure 9a), whereas strongly constrained structural elements typically exhibit wedge-shaped failures (Figure 9b). Phase-field simulations further verify that boundary constraints govern both crack topology and initiation timing. The results demonstrate that strong constraints require higher corrosion levels to trigger complex crack systems, while the combined effect of non-uniform corrosion and weak constraints significantly reduces cover resistance and transitions failure modes from single-crack penetration to wedge-shaped spalling.

### 3.2. Non-Uniformity and Reinforcement Distribution

**Figure 10:**
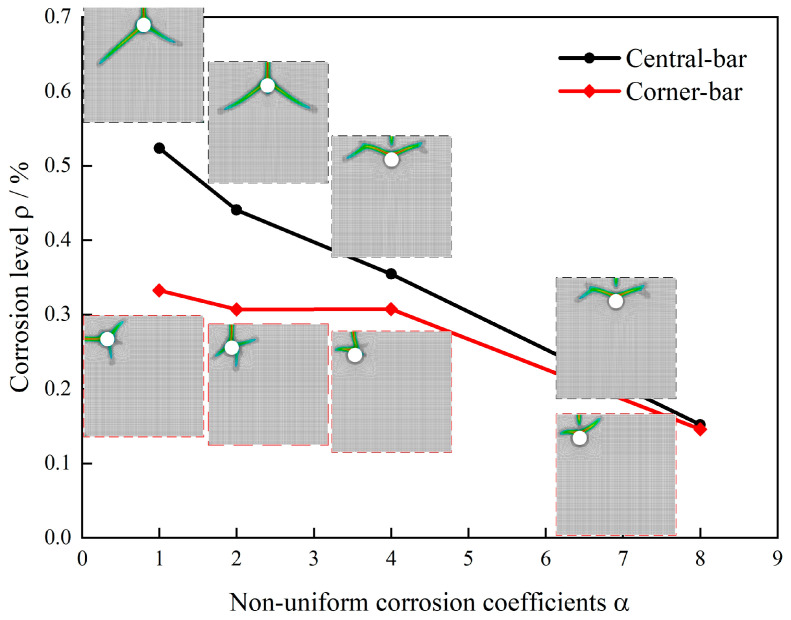
Crack propagation and corrosion levels under different reinforcement distributions.

Under consistent simply supported corner constraints and identical material parameters, this study compares the effects of central reinforcement placement versus corner reinforcement placement on crack propagation patterns and critical corrosion levels as the corrosion coefficient α increases from 1 to 8. Figure 10 presents crack evolution contours and ρ−α relationship curves for different reinforcement distributions under varying corrosion coefficients.

**Figure 11:**
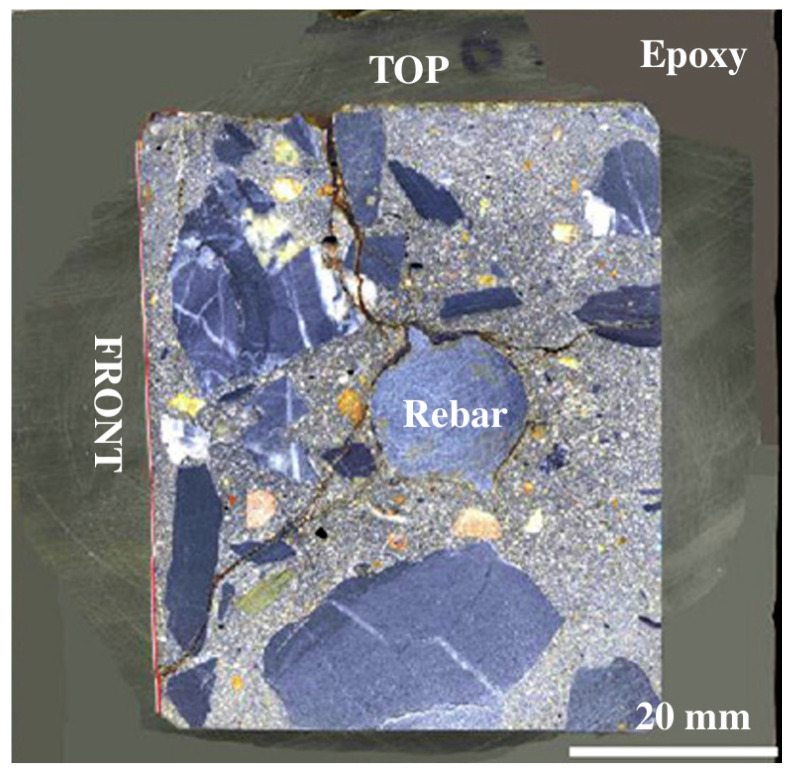
Crack propagation in corner-reinforced-concrete cover under non-uniform corrosion [57].

The reinforcement position significantly influences crack propagation behavior. For corner-reinforced specimens, the proximity of rebars to two free edges causes interfacial expansion to concentrate tensile strains at the corners. Even under uniform corrosion, diagonal splitting cracks develop along the corner bisectors, forming wedge-shaped cracks propagating inward from the corners. While increased corrosion non-uniformity modifies the wedge angle, the localized corner spalling characteristic persists—consistent with findings reported in the literature [57] (Figure 11) showing combined diagonal splitting and edge cracking failure modes. In contrast, centrally reinforced specimens exhibit more balanced constraint distribution, developing typical radial single-crack patterns under uniform corrosion, with wedge-shaped cracking emerging only as corrosion non-uniformity intensifies.

Critical corrosion level analysis reveals both configurations show monotonically decreasing ρ with increasing α, with corner reinforcement from 0.33% to 0.14%, mirroring the constraint condition study’s trend. Notably, corner-reinforced specimens demonstrate consistently lower critical corrosion levels than central-reinforced cases across all α values. This occurs because weaker localized constraints at corners promote earlier crack initiation with directional preference, while balanced constraints in central reinforcement delay cracking and produce more concentrated fracture patterns.

The comprehensive results demonstrate that reinforcement distribution influences crack resistance by modifying constraint intensity and directionality. Corner reinforcement exhibits “weak constraint–low threshold–dominant diagonal splitting” failure characteristics, whereas central reinforcement shows progressive “relatively strong constraint–high threshold–radial-to-wedge transition” failure modes.

### 3.3. Non-Uniformity and Corrosion Angle

**Figure 12:**
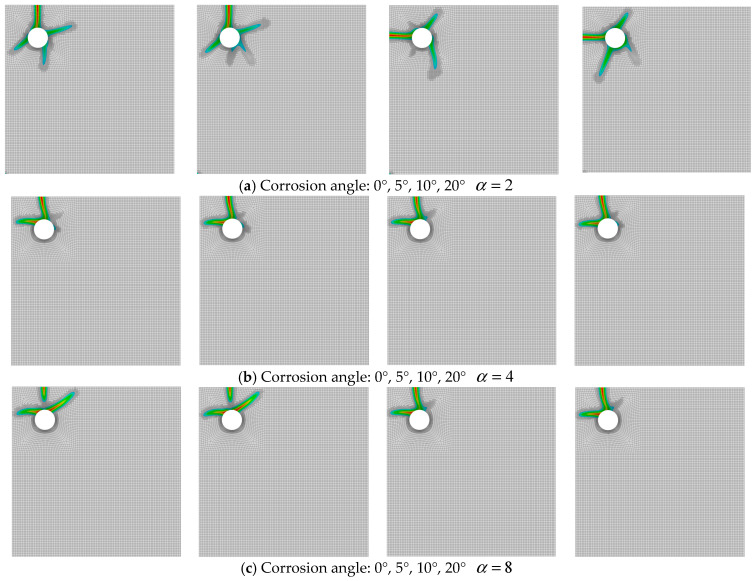
Crack propagation under different corrosion angles and coefficients.

**Figure 13:**
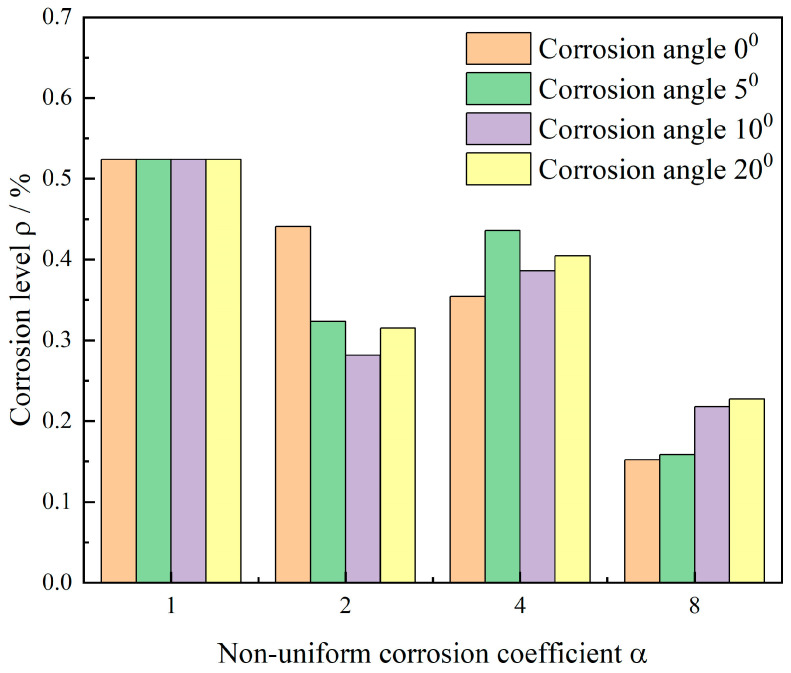
Steel corrosion levels under different corrosion angles and coefficients.

Under fixed conditions of simply supported corner constraints and eccentrically placed corner reinforcement, this study investigates the effects of different corrosion front orientations (φ = 0°, 5°, 10°, 20°) on crack propagation patterns and critical corrosion levels as the corrosion coefficient α increases from 2 to 8. Figure 12 and Figure 13, respectively, present crack evolution contours and ρ−α relationship curves for different corrosion angles under varying corrosion coefficients.

Regarding crack propagation characteristics, all angular conditions exhibited similar failure modes. As shown in Figure 12a–c, cracks consistently developed as wedge-shaped main fractures propagating from the corners inward, with initiation always occurring near the thinnest concrete cover. Notably, under identical α values, different angles produced minimal variations in crack topology, maintaining essentially consistent parameters including crack quantity, penetration range, and wedge opening angles. This indicates that under simply supported corner constraints, corrosion angle has limited influence on macroscopic crack morphology.

Corrosion level analysis revealed more nuanced patterns. The data in Figure 13 demonstrates that the effect of corrosion angle on critical corrosion levels shows a distinct corrosion coefficient dependency. At α=2, increasing angle reduced crack resistance, while at α = 4 or 8, larger angles improved corrosion failure resistance. Importantly, for given α values, angular variations caused relatively small fluctuations in corrosion levels without exhibiting clear monotonic trends.

The comprehensive results demonstrate that in simply supported corner constraint systems, the corrosion coefficient α serves as the dominant factor controlling crack initiation thresholds and failure morphology, while corrosion angle plays only a secondary role. Angular variations primarily cause minor deflection in crack propagation direction and exert slight modulation on critical corrosion levels.

### 3.4. Non-Uniformity and Structural Configuration

**Figure 14:**
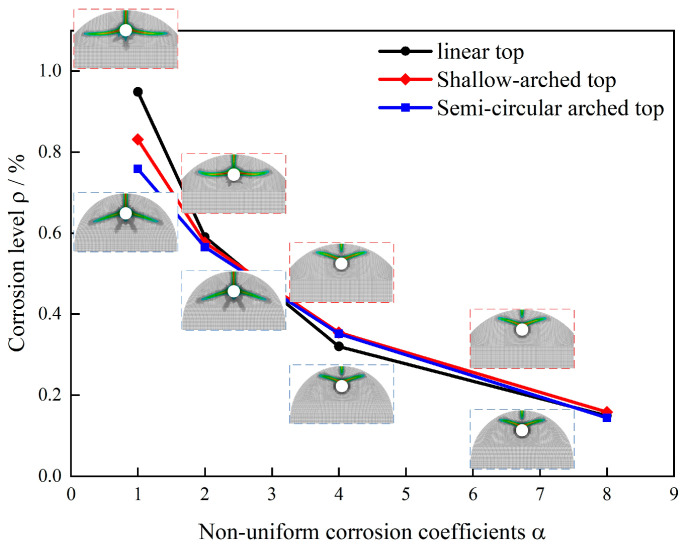
Crack propagation and corrosion levels under different structural configurations.

Under consistent material and numerical parameters, with centrally placed reinforcement, and fully fixed peripheral boundary constraints, this study compares crack evolution patterns and corresponding corrosion levels for three top surface geometries, as flat top, shallow arch top (R = 100 mm), and semi-circular arch top (R = 75 mm), under non-uniform corrosion coefficients α ∈ {1,2,4,8}. Figure 14 presents crack contours and ρ−α relationship curves for the three configurations.

All geometries exhibited similar crack initiation behavior. As shown in Figure 14, primary cracks consistently initiated directly above the mid-span reinforcement. Under uniform corrosion, cracks propagated radially along the shortest path, forming single fractures approximately perpendicular to the top surface. With increasing corrosion coefficient, cracks progressively deflected toward the peak corrosion side, developing wedge-shaped patterns with expanded surface spalling zones. Notably, arched specimens displayed curved secondary cracks due to boundary curvature effects, which is a key morphological distinction from flat-top specimens.

Analysis of corrosion levels revealed that while the critical corrosion levels of all three structural configurations decreased monotonically with increasing corrosion coefficients, notable differences existed. The flat top configuration exhibited a reduction from 0.95% to 0.15%, the shallow arch from 0.83% to 0.16%, and the semi-circular arch from 0.76% to 0.14%. Examination of the curve progression demonstrated that the semi-circular arch displayed the lowest crack resistance, followed by the shallow arch, with the flat top performing optimally. This behavior stems from variations in geometric constraints, where arched structures exhibit weaker self-restraint in their top regions, analogous to corner conditions, with the constraint reduction becoming more pronounced as curvature increases. Importantly, this geometric influence diminishes with enhanced corrosion non-uniformity, with the non-uniform distribution of corrosion becoming the dominant factor at higher corrosion coefficients.

These findings indicate that planar external surfaces can effectively delay cracking initiation under uniform or weakly non-uniform corrosion conditions. However, in environments with strongly non-uniform corrosion, the protective benefits of geometric configuration become limited. Comprehensive approaches incorporating material modifications and localized constraint reinforcement are therefore necessary to effectively control crack propagation.

### 3.5. Non-Uniformity and 3D Corrosion Morphology

**Figure 15:**
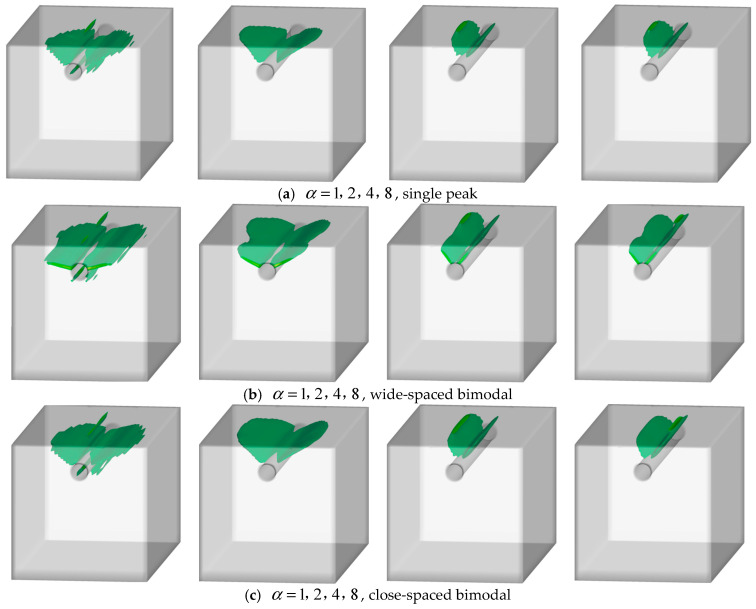
Crack propagation under different three-dimensional non-uniform corrosion morphologies (Green indicates cracks).

**Figure 16:**
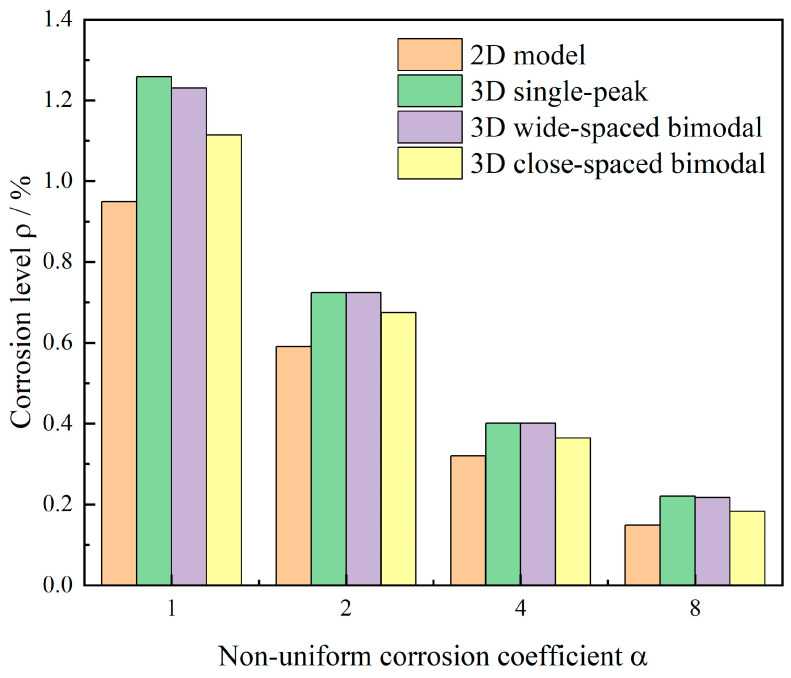
Steel corrosion levels under different three-dimensional non-uniform corrosion morphologies.

Under three-dimensional strong boundary constraints with identical non-uniform corrosion distribution applied circumferentially, this study investigated crack evolution and critical corrosion levels by varying only the longitudinal corrosion envelope G(z) among three patterns (single-peak, wide double-peak, and narrow double-peak) while examining α values of 1, 2, 4, and 8. Figure 15 presents three-dimensional phase-field contours and representative isosurfaces (d ≈ 0.4) showing crack patterns and damage zones, while Figure 16 displays ρ−α relationship curves compared with two-dimensional plane-strain models.

Regarding crack propagation characteristics, the three-dimensional models exhibited significant spatial localization effects. Under strong constraints from bottom and side surfaces, cracks consistently initiated first in the direction of minimum cover thickness before propagating toward the free top surface. Under single-peak envelope conditions (Figure 15a), damage concentrated in the mid-span region, forming a single dominant wedge-shaped crack. As the corrosion coefficient α increased, the wedge angle gradually decreased while the damaged zone shrank. The wide double-peak envelope (Figure 15b) generated two independent damage cores, producing separated wedge cracks and surface spalling patches with only weak connectivity in intermediate regions. The narrow double-peak envelope (Figure 15c) showed coupled damage zones forming continuous longitudinal spalling bands due to smaller peak spacing, resulting in the most extensive damage.

Corrosion level analysis revealed important dimensional effects (Figure 16). All cases showed monotonically decreasing critical corrosion levels with increasing α. The single-peak case decreased from 1.26% to 0.22%, the wide double-peak from 1.23% to 0.22%, and the narrow double-peak from 1.11% to 0.18%, compared with the two-dimensional case decreasing from 0.95% to 0.15%. This implies that, under the boundary conditions and parameter settings of this study, when three-dimensional longitudinal local non-uniformity is considered, the predicted cracking initiation threshold is higher compared to the two-dimensional idealized model, and the crack morphology more closely resembles the observed crack patterns. Among three-dimensional cases, single-peak envelopes performed best, followed by wide double-peak, with narrow double-peak showing the worst performance closest to the two-dimensional results. This suggests that greater spatial discretization of corrosion regions enables more complete longitudinal deformation release, improving crack resistance, while continuous corrosion zones strengthen constraints and reduce performance.

The study further found that although the influence of longitudinal envelope morphology diminished with increasing circumferential non-uniformity, significant differences persisted between two-dimensional and three-dimensional cases. This indicates that localized single-peak or discrete multi-peak corrosion distributions produce distinct short-band failure characteristics, while continuous banded corrosion distributions exhibit failure modes more closely resembling two-dimensional conditions.

## 4. Discussion

This study systematically quantified the effects of non-uniform corrosion, external constraints, reinforcement spatial position, corrosion angle, top surface geometry, and three-dimensional longitudinal corrosion envelopes on the corrosion-induced cracking of a concrete cover using the PF_CZM model. The characteristic displacement of reinforcement corrosion at cover failure was converted to corrosion level as a unified evaluation metric. The results demonstrate clear hierarchical differences in influencing factors. The corrosion coefficient emerged as the dominant factor, while boundary constraint intensity and three-dimensional longitudinal discreteness showed a comparable secondary influence. Reinforcement eccentricity exerted moderate effects, surface geometry exhibited relatively minor influence that further diminished under strong non-uniformity, and corrosion angle demonstrated only negligible impact.

The corrosion coefficient significantly reduced cracking thresholds and reshaped fracture topology, establishing itself as the most robust primary factor. Regardless of constraint intensity, critical corrosion levels decreased monotonically as α increased from 1 to 8. Under strong constraints, levels declined from approximately 0.95% to 0.15%, while weak constraints showed reduction from 0.52% to 0.15%, typically exceeding 70% reduction. Crack morphology transitioned from radial single-crack penetration to wedge-shaped cracking oriented toward peak corrosion zones, indicating local expansion gradients and circumferential tensile strain peaks govern crack deflection and surface spalling extent. These findings align with experimental observations reported in References [57,64].

External constraints substantially enhanced cracking resistance, though this benefit became partially offset under strong non-uniformity. At α = 1, fully fixed boundaries increased critical corrosion levels from 0.52% to 0.95% compared to simply supported corners, representing nearly 80% improvement. This difference rapidly diminished with increasing α, eventually becoming negligible. Morphologically, strong constraints favored wedge-shaped failures, suggesting earlier conversion of interfacial expansion into in-plane tensile stresses forming tension–shear composites, while weak constraints tended toward radial penetration along the shortest cover path. Engineering measures like circumferential confinement or external restraints can elevate cracking thresholds, though their effectiveness becomes limited under strongly non-uniform conditions such as unilateral corrosion.

Reinforcement position exerted moderate influence by altering effective constraints and proximity to free edges. Eccentric corner placement adjacent to two free edges caused stress concentration, yielding critical corrosion levels of 0.33% at α = 1, significantly lower than 0.52% for central placement. At α = 8, these values converged to 0.14% and 0.15%, respectively. Corner reinforcement typically developed combined diagonal splitting and edge cracking, whereas central placement showed progressive transition from radial single-crack to wedge-shaped failure. Design practices should avoid placing main reinforcement near corners, instead applying enhanced constraints and thicker covers when necessary.

Corrosion angle φ exerted minimal influence on macroscopic crack morphology, primarily causing slight propagation direction deflection. Quantitatively, φ variations between 0° and 20° produced minor modulation that coupled with α. At α = 2, increasing φ slightly reduced crack resistance, while at α = 4 or 8 it provided marginal improvement, showing no monotonic trend overall.

Top surface geometry demonstrated lesser influence than constraints or position, though it retained engineering significance under weak non-uniformity. Under identical constraints and reinforcement placement, flat, shallow arch, and semi-circular arch tops showed declining critical corrosion levels with increasing corrosion coefficient, ranked as flat top best, shallow arch intermediate, and semi-circular arch worst. At α = 1, semi-circular arches showed approximately 20% lower thresholds than flat tops, with shallow arches being 15% lower. These differences became negligible at α = 8. Arched tops exhibited greater geometric freedom at mid-span with weaker equivalent constraints, resembling corner effects and promoting wedge-shaped cracking and surface spalling. During carbonation-dominated or environmentally uniform early stages, flat surfaces with uniform covers effectively delay cracking, though geometric optimization provides diminishing returns under strong non-uniformity.

Under the boundary conditions and parameter settings adopted in this study, accounting for three-dimensional longitudinal corrosion non-uniformity results in a higher predicted cracking initiation threshold relative to the two-dimensional idealized model. Compared to two-dimensional plane-strain results decreasing from 0.95% to 0.15%, three-dimensional models showed single-peak envelopes decreasing from 1.26% to 0.22%, wide double-peak from 1.23% to 0.22%, and narrow double-peak from 1.11% to 0.18%. Three-dimensional degrees of freedom and longitudinal discreteness provided additional strain relief channels, making two-dimensional assessments conservative. Greater peak discreteness improved crack resistance, while reduced peak spacing producing near-continuous corrosion zones yielded results closer to two-dimensional cases with increased longitudinal spalling band formation. Differences among envelope types diminished with increasing α, though three-dimensional advantages persisted.

Comprehensive comparison established the following influence hierarchy: non-uniformity α showed the strongest effect, typically reducing critical levels by 70–85%; boundary constraints and three-dimensional discreteness ranked next, increasing thresholds by 30–80% with effects weakening at higher α; reinforcement eccentricity reduced thresholds by 30–40% at α = 1, narrowing to 5–10% at α = 8; surface geometry showed a minor influence, reducing thresholds by 10–20% at α = 1 with a negligible difference at α = 8; corrosion angle exerted only slight non-monotonic effects. Factor coupling occurred, with strong non-uniformity diminishing beneficial effects of constraints and geometry. Design and assessment should prioritize mitigating non-uniform corrosion sources like unilateral chloride penetration and wet–dry cycling variation, while combining enhanced constraints and optimized covers at vulnerable regions for maximum durability benefits.

To contextualize the findings of this study, Table 3 provides a concise comparison with representative cited works, summarizing their main conclusions and highlighting the differences in methodology, scope, and the unified threshold metric employed herein.

Several assumptions and limitations remain. Corrosion expansion was represented through equivalent normal displacement without explicit consideration of rust product mechanics–transport–volume evolution coupling or interfacial bond degradation. Multi-rebar interaction and load–corrosion coupling were not included. Future work could couple electrochemical-transport processes with phase-field fracture, incorporate variable expansion ratios and interfacial constitutive laws, extend to multiple rebars and loaded members, and validate through multiscale experimentation to improve reliability and applicability of critical corrosion level predictions. The expansion coefficient of corrosion products in this study is treated as a fixed value. Future research could consider variations in the equivalent expansion coefficient under different conditions and incorporate experimental data for calibration, thereby enhancing the model’s applicability and realism.

## 5. Conclusions

Based on the PF_CZM phase-field model, this study systematically evaluates the effects of non-uniform corrosion, external restraint, rebar spatial position, corrosion angle, top-surface geometry, and three-dimensional longitudinal corrosion envelopes on the rust-expansion cracking of a concrete cover. A unified quantitative metric is adopted: the corrosion level converted from the characteristic corrosion-induced displacement of the rebar at the moment of cover failure. The main conclusions are as follows.

(1) External restraint increases the resistance of reinforced concrete to corrosion-induced damage; strong restraint requires a larger corrosion level to initiate cracking and more readily leads to multiple cracking or spalling. Quasi-free boundaries initiate earlier and are predominantly characterized by a single crack.
(2) Increasing circumferential non-uniformity lowers the crack initiation threshold. When three-dimensional longitudinal localization is considered, the predicted initiation threshold is higher than that from a two-dimensional idealization, and the simulated crack morphology is closer to observations.
(3) Rebar eccentricity reduces the initiation threshold and deflects cracks toward edges and corners. Under weak non-uniformity, the top-surface geometry affects crack resistance, with a flat surface outperforming an arched surface; this effect diminishes under strong non-uniformity. The corrosion angle mainly alters crack orientation and has only a minor influence on the threshold.
(4) Regarding the influence on the initiation threshold, the factors rank, in descending order: non-uniformity; boundary restraint and three-dimensional localization; rebar eccentricity. The effect of top-surface geometry is smaller, and corrosion angle is the weakest.
(5) For engineering practice, priority should be given to mitigating sources of non-uniform corrosion. In early stages or weakly non-uniform environments, a flat exterior surface, centered reinforcement, and circumferential stirrups are recommended to enhance restraint; when reinforcement is placed near corners, the cover thickness should be increased and local restraint strengthened; for critical regions, three-dimensional analysis is recommended to avoid the bias of two-dimensional assessment.

Future work will include long-term or full-scale experiments, extension to multi-bar and mixed-restraint scenarios, and incorporation of bond deterioration and stochastic variability to develop reliability-based thresholds for crack initiation.

## Figures and Tables

**Table 1:** Calculation parameters of PF_CZM model.

Model	$c_{a}$	$a_{1}$	$a_{2}$	$a_{3}$	r	ξ
PF_CZM	π	$\frac{4G_{c}\cdot E_{o}}{\pi l\cdot f_{t}^{2}}$	1.3868	0.6567	2.0	2.0

**Table 2:** Calculation parameters of plain concrete.

Parameter	Symbol	Value
Elastic modulus	$E_{0}$ (GPa)	30.0
Poisson’s ratio	v	0.167
Regularization width	*l* (mm)	4.0
Griffith’s constant	*G_c_* (N·mm^−1^)	100
Tensile strength	$f_{t}$ (MPa)	2.5
Compressive strength	$f_{c}$ (MPa)	29.1

**Table 3:** Concise comparative summary.

Author (Year) [Ref]	Main Result	Difference from This Article
Jang & Oh (2010) [64]	Non-uniform corrosion triggers earlier cracking and shortens service life.	We show the critical corrosion level decreases monotonically with the non-uniformity coefficient and clarify coupled effects on thresholds and crack patterns.
Zhao et al. (2011) [57]	Uses a Gaussian description to distinguish damage from uniform vs. non-uniform corrosion.	We use a power law for transverse non-uniformity and a Gaussian for longitudinal non-uniformity.
Wei et al. (2021) [33]	Hydro-chemo-mechanical phase-field shows fracture toughness and permeability strongly affect crack evolution.	We model mechanically driven cracking via equivalent interface displacement, analyze multiple factors, and report quantitative threshold trends.
Freddi & Mingazzi (2021) [32]	Phase-field simulation of cover cracking in beams under carbonation.	We quantify both carbonation and chloride corrosion, considering cover restraint and geometry.
Fang et al. (2023) [34]	A multi-phase-field framework reproduces non-uniform corrosion-induced cracking, consistent with experiments.	We propose a unified corrosion morphology function, rank factor influence, and give detailed recommendations; results agree with experiments.
Wang et al. (2022) [16]	3D FE shows non-uniform attack causes earlier serviceability limits and more severe cracking; stirrups can raise the cracking threshold.	Our improved FE model shows strong restraint raises the cracking threshold; 3D models give higher thresholds than 2D.
Biswas et al. (2020) [4]	Non-uniform corrosion degrades structural performance earlier than uniform corrosion.	We quantify impacts of non-uniformity, restraint, geometry, and 3D on thresholds and crack patterns (ranking: non-uniformity > restraint ≈ 3D > eccentricity > geometry > corrosion angle).

## Data Availability

The raw data supporting the conclusions of this article will be made available by the authors on request.
